# Multilocus Sequence Typing of Pathogenic Treponemes Isolated from Cloven-Hoofed Animals and Comparison to Treponemes Isolated from Humans

**DOI:** 10.1128/AEM.00025-16

**Published:** 2016-07-15

**Authors:** Simon R. Clegg, Stuart D. Carter, Richard J. Birtles, Jennifer M. Brown, C. Anthony Hart, Nicholas J. Evans

**Affiliations:** a Department of Infection Biology, Institute of Infection and Global Health, School of Veterinary Science, University of Liverpool, Liverpool, United Kingdom; b School of Environment and Life Sciences, University of Salford, Salford, United Kingdom; c Department of Medical Microbiology, University of Liverpool, Liverpool, United Kingdom; Washington State University

## Abstract

Treponema species are implicated in many diseases of humans and animals. Digital dermatitis (DD) treponemes are reported to cause severe lesions in cattle, sheep, pigs, goats, and wild elk, causing substantial global animal welfare issues and economic losses. The fastidiousness of these spirochetes has previously precluded studies investigating within-phylogroup genetic diversity. An archive of treponemes that we isolated enabled multilocus sequence typing to quantify the diversity and population structure of DD treponemes. Isolates (*n* = 121) were obtained from different animal hosts in nine countries on three continents. The analyses herein of currently isolated DD treponemes at seven housekeeping gene loci confirm the classification of the three previously designated phylogroups: the Treponema medium, Treponema phagedenis, and Treponema pedis phylogroups. Sequence analysis of seven DD treponeme housekeeping genes revealed a generally low level of diversity among the strains within each phylogroup, removing the need for the previously used “-like” suffix. Surprisingly, all isolates within each phylogroup clustered together, regardless of host or geographic origin, suggesting that the same sequence types (STs) can infect different animals. Some STs were derived from multiple animals from the same farm, highlighting probable within-farm transmissions. Several STs infected multiple hosts from similar geographic regions, identifying probable frequent between-host transmissions. Interestingly, T. pedis appears to be evolving more quickly than the T. medium or T. phagedenis DD treponeme phylogroup, by forming two unique ST complexes. The lack of phylogenetic discrimination between treponemes isolated from different hosts or geographic regions substantially contrasts with the data for other clinically relevant spirochetes.

**IMPORTANCE** The recent expansion of the host range of digital dermatitis (DD) treponemes from cattle to sheep, goats, pigs, and wild elk, coupled with the high level of 16S rRNA gene sequence similarity across hosts and with human treponemes, suggests that the same bacterial species can cause disease in multiple different hosts. This multilocus sequence typing (MLST) study further demonstrates that these bacteria isolated from different hosts are indeed very similar, raising the potential for cross-species transmission. The study also shows that infection spread occurs frequently, both locally and globally, suggesting transmission by routes other than animal-animal transmission alone. These results indicate that on-farm biosecurity is important for controlling disease spread in domesticated species. Continued surveillance and vigilance are important for ascertaining the evolution and tracking any further host range expansion of these important pathogens.

## INTRODUCTION

Only rarely do we encounter infectious agents spreading rapidly through different animal populations and causing substantial and varied disease manifestations in a wide variety of hosts. Classically, digital dermatitis (DD) is a disease of dairy cattle, first seen in 1974 and known to cause severe lameness (1). DD is now considered endemic in dairy cattle in many countries worldwide, and it is a serious animal welfare issue on farms. Economic impacts of the disease, due to reductions in milk yields and reproductive performance, have been estimated at $190 million per annum in the United States alone (2).

A considerable body of evidence identifies specific Treponema species as the etiological agents of DD. More recently, since it was first reported in 1997, DD has spread through sheep farms in the United Kingdom (3), and in very recent times, it has been reported for goats in the United Kingdom (4). In these two host species, the same treponeme phylotypes associated with cattle DD are consistently identified in foot lesions, and they result in severe clinical outcomes that are very difficult to treat. The DD treponemes were recently associated with foot lesions causing lameness in wild American elk (Cervus elaphus) (5). Reports show that DD treponemes can be isolated from and associated with porcine ear and shoulder skin lesions (6–8). In humans, Treponema spp. are considered to be responsible for periodontal disease and syphilis. Interestingly, while oral treponemes are reported to be closely related to DD treponemes, the agent of syphilis is substantially different (9, 10).

To date, five major phylotypes of treponemes have been highly associated with DD (9–12). Three of these DD-associated phylogroups have repeatedly been isolated from animals symptomatic for DD and have been designated coherent groups on the basis of genotypic and phenotypic characterizations (10, 13–16). Previous studies identified the culturable DD treponemes as highly similar to human periodontal and genitourinary treponemes, based on their 16S rRNA genes, and due to a lack of additional data, this led to assignment of a “-like” suffix (10, 13–15). Contrastingly, a recent study suggested the removal of the “-like” suffix for bovine Treponema phagedenis isolates (17). The three cultivable treponemes have been grouped into the Treponema medium/Treponema vincentii, Treponema phagedenis, and Treponema putidum/Treponema denticola DD spirochete phylogroups (10, 14–16). Subsequently, the latter phylogroup was designated a novel species, i.e., Treponema pedis (13).

The fastidious nature of these microorganisms and the difficulty of obtaining pure treponemes have previously led to a dearth of isolates. However, our bacterial culture developments have enabled the accumulation of an archive of treponeme isolates which may now allow comparative analyses to investigate their genetic relationships.

Sequencing of the 16S rRNA gene demonstrated clear differences between the three commonly isolated phylogroups of DD treponemes, with these groups sharing only 90.1% to 92.3% 16S rRNA gene sequence identity, and they are therefore considered separate phylogroups/species (10). However, little 16S rRNA gene sequence variation within phylogroups has been identified, with no notable variation between different treponemes within a phylogroup isolated from different hosts (4, 5, 18). Other studies analyzed a number of genetic loci, including intragenic spacer regions (ISR1 and ISR2) and *flab2*, but this did not allow for isolate discrimination beyond that observed using 16S rRNA gene sequence comparisons (10, 14, 19). To further investigate the DD treponemes, additional genotyping studies are required to allow intraphylogroup discrimination.

Multilocus sequence typing (MLST) or multilocus sequence analysis (MLSA; differs in the analyses used) schemes have now been described for a range of spirochetes (20–24) and serve as key frameworks for the phylogeny and taxonomy of these taxa. Given recent rapid expansions in the host ranges of the DD treponemes, it is particularly timely to determine if the same bacteria infect multiple animal species or whether several different host-specific genotypes exist. The aim of this study was to design an MLST protocol for the three important phylogroups of DD treponemes which can be grown in culture and to investigate cross-host-species disease transmission. Such comprehensive molecular genetic analyses of the different treponemes isolated from a variety of hosts and geographic regions should ascertain their similarities and identify relevant relationships between human- and animal-pathogenic treponemes.

This report describes the MLST classification of 121 fastidious treponeme isolates, the vast majority of which were obtained from animal tissues during the past 10 years. The data reveal interesting insights into the transmission of disease between host species on various spatial scales (including within farms, within countries, and more globally) and into the role of treponeme evolution in such processes.

## MATERIALS AND METHODS

### Bacterial taxonomy.

In the majority of previous studies, a “-like” suffix has been used for bovine DD T. medium and T. phagedenis spirochetes, based on their close similarity to human treponeme relatives (using 16S rRNA gene analysis). For clarity, this study proposes removal of the “-like” suffix, with subsequent references to the “T. medium phylogroup” and the “T. phagedenis phylogroup,” as previously suggested for bovine DD T. phagedenis (17). Each phylogroup includes isolates which share more than 97% 16S rRNA gene sequence identity to what are considered the representative strains of the three different phylogroups, namely, T19, T320A, and T3552B^T^. The 97% 16S rRNA gene sequence identity criterion has been used frequently in previous taxonomic assignments of bacterial and, specifically, treponemal species (10, 13, 25–27).

### Treponeme isolates.

In this study, 121 isolates were investigated; 116 isolates were regrown, passaged, and purified to ensure that a pure isolate of each was used for genotyping. Forty-eight of these are previously undescribed isolates.

All 116 isolates were grown on fastidious anaerobe agar (FAA) plates supplemented with 5% defibrinated sheep blood and antibiotics (10), from which single colonies were picked into OTEB tubes as previously described (10). The provenances of isolates are presented in Tables 1 to 3. The three U.S. cattle isolates were a kind gift from Richard Walker, and human isolates were obtained from the American Type Culture Collection (ATCC) and the Collection of the Institut Pasteur (CIP).

**TABLE 1:** T. medium phylogroup (DD1) isolate details^*a*^

Isolate name	Host from which isolate was obtained	Yr of isolation	Farm and geographic provenance	ST	MLST allele							16S rRNA gene GenBank accession no.	Reference
					*groEL*	*recA*	*glpK*	*adk*	*gdh*	*pyrG*	*rplB*		
T19	Dairy	2003	Farm A, Merseyside, England	1	1	1	1	1	1	1	1	EF061249	10
G12F2	Sheep	2013	Farm B, Conwy, Wales	1	1	1	1	1	1	1	1	KP063172	18
ST27	Sheep	2013	Farm C, Conwy, Wales	1	1	1	1	1	1	1	1	KR025808	This study
g1F7c5	Sheep	2013	Farm C, Conwy, Wales	1	1	1	1	1	1	1	1	KP063152	18
g1F9c27	Sheep	2013	Farm C, Conwy, Wales	1	1	1	1	1	1	1	1	KP063153	18
g16F2	Sheep	2013	Farm B, Conwy, Wales	1	1	1	1	1	1	1	1	KP063174	18
T56	Dairy	2003	Farm A, Merseyside, England	1	1	1	1	1	1	1	1	EF061251	10
T54	Dairy	2003	Farm A, Merseyside, England	1	1	1	1	1	1	1	1	EF061250	10
T184Y (RLUH-1)	Dairy	2003	Farm A, Merseyside, England	1	1	1	1	1	1	1	1	AY387410	10
T18A	Dairy	2003	Farm A, Merseyside, England	1	1	1	1	1	1	1	1	EF061252	10
T35B1	Dairy	2003	Farm A, Merseyside, England	1	1	1	1	1	1	1	1	KR025809	This study
ST12	Sheep	2013	Farm B, Conwy, Wales	1	1	1	1	1	1	1	1	KR025810	This study
MED1985 AG 3	Dairy	1994	Farm D, California, USA	1	1	1	1	1	1	1	1	KR025853	15
T200BA2	Dairy	2004	Farm E, Shropshire, England	1	1	1	1	1	1	1	1	KR025811	This study
T. medium ATCC 700293	Human	1972	Japan	2	2	2	2	2	2	2	2	D85437	50
7.45 G	Goat	2013	Farm F, Lancashire, England	3	1	1	3	4	1	1	3	KR025812	This study
T136E	Dairy	2004	Farm G, Shropshire, England	4	1	1	1	3	1	1	1	FJ204242	55
T52B	Dairy	2004	Farm G, Shropshire, England	5	1	1	1	1	3	1	1	FJ204241	55
OV11F	Sheep	2009	Farm H, Gloucestershire, England	6	1	1	1	1	1	3	1	KR025813	This study
EL023 aR	Elk	2013	Washington State, USA	6	1	1	1	1	1	3	1	KM586669	5
G2S2R	Sheep	2009	Farm I, Cheshire, England	6	1	1	1	1	1	3	1	KP063164	18
T200BA1	Dairy	2004	Farm G, Shropshire, England	7	1	1	1	4	1	1	1	KR025814	This study
EL022R	Elk	2013	Washington State, USA	7	1	1	1	4	1	1	1	KM586668	5
DD3F (1)	Dairy	2009	Farm J, Merseyside, England	7	1	1	1	4	1	1	1	KR025815	This study
2c	Beef	2012	Farm K, Gloucestershire, England	7	1	1	1	4	1	1	1	KP859546	This study
2D	Beef	2012	Farm K, Gloucestershire, England	7	1	1	1	4	1	1	1	KP859544	This study
T296	Dairy	2004	Farm L, Cheshire, England	8	1	1	1	1	1	1	3	KR025816	This study
T380	Dairy	2004	Farm J, Merseyside, England	8	1	1	1	1	1	1	3	KR025817	This study
T3551	Dairy	2004	Farm J, Merseyside, England	8	1	1	1	1	1	1	3	KR025818	This study
T3202F	Dairy	2004	Farm J, Merseyside, England	8	1	1	1	1	1	1	3	KR025819	This study
3E	Beef	2012	Farm K, Gloucestershire, England	9	1	1	1	4	1	3	1	KP859545	This study
G1OV11	Sheep	2009	Farm H, Gloucestershire, England	9	1	1	1	4	1	3	1	KP063154	18
EL024 R	Elk	2013	Washington State, USA	10	1	1	1	4	1	3	3	KM586673	5
T vincentii OMZ 838	Human	1998	China	11	3	3	4	5	4	4	4	CP009227	28

a Including allelic arrangements (DNA) for the 34 isolates analyzed for the T. medium phylogroup. GenBank accession numbers for the 16S rRNA gene and papers in which the isolates are previously referenced are also shown.

**TABLE 2:** Isolation details, with allelic arrangements (DNA), for the 70 isolates from the T. phagedenis phylogroup (DD2) analyzed as part of this study^*a*^

Sample	Origin	Yr of isolation	Farm and geographic provenance	ST	MLST allele							16S rRNA gene GenBank accession no.	Reference
					*groEL*	*recA*	*glpK*	*adk*	*gdh*	*pyrG*	*rplB*		
T320A	Dairy	2004	Farm J, Merseyside, England	1	1	1	1	1	1	1	1	EF061261	10
G2F3	Sheep	2013	Farm B, Conwy, Wales	1	1	1	1	1	1	1	1	KP063156	18
EL024 F	Elk	2013	Washington State, USA	1	1	1	1	1	1	1	1	KM586672	5
EL022 F	Elk	2013	Washington State, USA	1	1	1	1	1	1	1	1	KM586667	5
EL023 F	Elk	2013	Washington State, USA	2	1	9	1	1	1	1	1	KM586670	5
G187	Dairy	2004	Farm M, Gloucestershire, England	2	1	9	1	1	1	1	1	EF061266	10
G23F1	Sheep	2013	Farm N, Anglesey, Wales	2	1	9	1	1	1	1	1	KP063178	18
1498 MED AG	Dairy	1994	Farm D, California, USA	2	1	9	1	1	1	1	1	KR025851	15
T122A	Dairy	2005	Farm L, Cheshire, England	2	1	9	1	1	1	1	1	FJ204238	55
C2R (1)	Sheep	2009	Farm I, Cheshire, England	3	3	9	1	1	4	1	1	KR025821	This study
C2F	Sheep	2009	Farm I, Cheshire, England	3	3	9	1	1	4	1	1	KR025822	This study
10C	Beef	2012	Farm K, Gloucestershire, England	3	3	9	1	1	4	1	1	KP859543	This study
C2RA	Dairy	2009	Farm L, Cheshire, England	3	3	9	1	1	4	1	1	KR025820	This study
T167LAB2	Dairy	2003	Farm L, Cheshire, England	3	3	9	1	1	4	1	1	EF061253	10
T100A	Dairy	2005	Farm L, Cheshire, England	3	3	9	1	1	4	1	1	FJ204239	55
T323C F1	Dairy	2004	Farm A, Merseyside, England	4	3	9	1	1	5	1	1	EF061263	10
T2723	Dairy	2004	Farm A, Merseyside, England	4	3	9	1	1	5	1	1	FJ204237	55
T2721A	Dairy	2004	Farm A, Merseyside, England	5	3	9	1	1	5	1	2	EF061260	10
DD3F (2)	Dairy	2009	Farm J, Merseyside, England	6	1	9	1	2	4	1	1	KR025823	This study
T. phagedenis Reiter	Human	1926	Germany	7	3	8	4	3	4	3	1	KR025824	51
G169A	Dairy	2004	Farm M, Gloucestershire, England	8	3	9	1	1	2	1	1	EF061265	10
ST27	Sheep	2013	Farm B, Conwy, Wales	9	3	9	1	1	1	1	1	KR025825	This study
G26F1	Sheep	2013	Farm O, Denbighshire, Wales	9	3	9	1	1	1	1	1	KP063180	18
DD4F	Dairy	2009	Farm J, Merseyside, England	9	3	9	1	1	1	1	1	KR025826	This study
S4R	Sheep	2009	Farm I, Cheshire, England	9	3	9	1	1	1	1	1	KR025827	This study
T136	Dairy	2004	Farm G, Shropshire, England	10	3	9	1	2	3	1	1	EF061255	10
T119A	Dairy	2004	Farm G, Shropshire, England	10	3	9	1	2	3	1	1	EF061256	10
T354B	Dairy	2004	Farm L, Cheshire, England	10	3	9	1	2	3	1	1	EF061259	10
T35	Dairy	2004	Farm J, Merseyside, England	10	3	9	1	2	3	1	1		This study
SL4	Sheep	2013	Farm N, Anglesey, Wales	11	3	2	1	1	4	1	1	KR025828	This study
G2S4F	Sheep	2009	Farm I, Cheshire, England	11	3	2	1	1	4	1	1	KP063166	18
SL2	Sheep	2013	Farm N, Anglesey, Wales	12	1	2	1	1	4	1	1	KR025829	This study
G2SL1	Sheep	2013	Farm N, Anglesey, Wales	12	1	2	1	1	4	1	1	KP063167	18
G10JD	Goat	2013	Farm F, Lancashire, England	13	1	1	1	1	4	1	1	KJ206532	4
T645C3	Dairy	2004	Farm A, Merseyside, England	14	3	1	1	1	5	1	1	FJ204236	55
6LD	Beef	2013	Farm P, Anglesey, Wales	15	3	1	1	1	4	1	1	KP859539	This study
2LC	Beef	2013	Farm P, Anglesey, Wales	15	3	1	1	1	4	1	1	KP859540	This study
G2S1F	Sheep	2009	Farm Q, Cheshire, England	16	2	1	1	1	4	1	1	KP063163	18
S2321	Sheep	2009	Farm Q, Cheshire, England	16	2	1	1	1	4	1	1	KR025830	This study
S5R	Sheep	2009	Farm Q, Cheshire, England	16	2	1	1	1	4	1	1	KR025831	This study
G2S3R1	Sheep	2009	Farm Q, Cheshire, England	16	2	1	1	1	4	1	1	KP063165	18
S32R	Sheep	2009	Farm I, Cheshire, England	16	2	1	1	1	4	1	1	KR025832	This study
S3R	Sheep	2009	Farm I, Cheshire, England	16	2	1	1	1	4	1	1	KR025833	This study
11A	Beef	2012	Farm K, Gloucestershire, England	17	3	9	1	1	2	1	1	KP859541	This study
1A	Beef	2012	Farm K, Gloucestershire, England	17	3	9	1	1	2	1	1	KP750188	This study
T296A	Dairy	2004	Farm L, Cheshire, England	17	3	9	1	1	2	1	1	EF061258	10
T257	Dairy	2004	Farm L, Cheshire, England	17	3	9	1	1	2	1	1	EF061257	10
T380 A2F45	Dairy	2004	Farm A, Merseyside, England	17	3	9	1	1	2	1	1	EF061262	10
T. phagedenis ATCC Kazan 8	Human	1984	Russia	18	3	6	4	3	2	3	1	KR025835	52
T. phagedenis CIP	Human	1962	France	19	3	5	3	3	2	2	1	KR025834	10
P	Dairy	2000	Farm A, Cheshire, England	20	3	9	1	2	2	1	1	KR025836	This study
K	Dairy	2000	Farm A, Cheshire, England	20	3	9	1	2	2	1	1	KR025837	This study
DD2R	Dairy	2009	Farm J, Merseyside, England	21	3	9	1	1	1	1	1	KR025838	This study
DD2F	Dairy	2009	Farm J, Merseyside, England	22	1	9	1	2	1	1	1	KR025839	This study
EL022a F	Elk	2013	Washington State, USA	23	1	7	1	1	1	1	1	KM586666	5
W35	Dairy	2004	Farm L, Cheshire, England	24	1	9	1	1	1	1	2	EF061264	10
DD1R	Dairy	2009	Farm J, Merseyside, England	25	1	9	2	1	1	1	1	KR025840	This study
DD5F	Dairy	2009	Farm J, Merseyside, England	25	1	9	2	1	1	1	1	KR025841	This study
T200	Dairy	2004	Farm G, Shropshire, England	26	3	4	1	1	1	1	1	FJ204240	55
T52	Dairy	2004	Farm G, Shropshire, England	27	3	1	1	1	1	1	1	EF061254	55
3F2	Sheep	2014	Farm N, Anglesey, Wales	27	3	1	1	1	1	1	1	KR025842	This study
T116B	Dairy	2005	Farm A, Merseyside, England	28	1	3	1	1	1	1	1	FJ204237	55
G2SL5	Sheep	2013	Farm N, Anglesey, Wales	29	1	2	1	1	1	1	1	KP063168	18
ST25	Sheep	2013	Farm B, Conwy, Wales	30	1	2	1	1	2	1	1	KR025843	This study
ST26	Sheep	2013	Farm B, Conwy, Wales	31	1	2	1	1	2	1	1	KR025844	This study
G2ST24	Sheep	2013	Farm B, Conwy, Wales	31	1	2	1	1	2	1	1	KP063169	18
DD1F	Dairy	2009	Farm J, Merseyside, England	32	1	9	2	1	2	1	1	KR025845	This study
T. phagedenis 4A	Dairy	Unknown	Iowa, USA	33	3	9	1	1	4	1	3	AQCF00000000	17
T. phagedenis F0421	Human	Unknown	USA	34	3	7	5	3	4	3	1	NZ_AEFH00000000	17
T. phagedenis V1	Dairy	Unknown	Sweden	35	1	9	1	1	2	1	1	CDNC01000001–CDNC01000051	53

a GenBank accession numbers for the 16S rRNA gene and papers in which the isolates are previously referenced are also shown.

**TABLE 3:** Isolation details, with allelic arrangements (DNA), for the 17 isolates from the T. pedis phylogroup (DD3) analyzed as part of this study^*a*^

Sample	Origin	Yr of isolation	Farm and geographic provenance	ST	MLST allele							16S rRNA gene GenBank accession no.	Reference
					*groEL*	*recA*	*glpK*	*adk*	*gdh*	*pyrG*	*rplB*		
T3552B^T^	Dairy	2004	Merseyside, England	1	1	1	1	1	1	1	1	EF061268	10
T136P2	Dairy	2004	Farm E, Shropshire, England	1	1	1	1	1	1	1	1	FJ204243	13
G3ST1	Sheep	2014	Farm R, Shropshire, England	2	4	5	4	5	5	5	4	KP063171	18
G3S4S	Sheep	2014	Farm R, Shropshire, England	2	4	5	4	5	5	5	4	KP063170	18
G3T1	Sheep	2014	Farm R, Shropshire, England	2	4	5	4	5	5	5	4	KR025846	This study
G3T7	Sheep	2014	Farm R, Shropshire, England	2	4	5	4	5	5	5	4	KR025847	This study
G9JD	Goat	2013	Farm F, Lancashire, England	2	4	5	4	5	5	5	4	KJ206531	4
G2JD	Goat	2013	Farm F, Lancashire, England	3	4	4	4	5	5	5	4	KJ206528	4
9185 Med Ag 2	Dairy	1994	Farm D, California, USA	4	2	2	2	2	2	2	3	KR025852	15
T184F2	Dairy	2003	Farm A, Merseyside, England	5	3	6	3	4	4	4	3	KR025848	This study
T18D2 (T18B)	Dairy	2003	Farm A, Merseyside, England	5	3	6	3	4	4	4	3	EF061270	10
DD3F (3)	Dairy	2009	Farm J, Merseyside, England	5	3	6	3	4	4	4	3	KR025849	This study
T354A	Dairy	2004	Farm L, Cheshire, England	5	3	6	3	4	4	4	3	EF061267	10
G819CB	Dairy	2004	Farm M, Gloucestershire, England	5	3	6	3	4	4	4	3	EF061269	10
Ovine (G179)	Sheep	2006	Farm S, Northern Ireland	5	3	6	3	4	4	4	3	AF363634	54
T3551C	Dairy	2004	Farm A, Merseyside, England	6	1	1	1	5	1	1	1	KR025850	This study
T. pedis T A4	Pig	2013	Sweden	7	1	3	2	3	3	3	2	CP004120	7

a GenBank accession numbers for the 16S rRNA gene and papers in which the isolates are previously referenced are also shown.

In addition, the DNA sequences of the following five samples available in GenBank were used in the study: T. vincentii OMZ 838 (accession no. CP009227) (28); a T. pedis strain (accession no. CP004120) isolated from a pig (7); two shotgun-sequenced T. phagedenis isolates from cattle, one from Iowa (17) and one from Sweden (accession no. AQCF00000000 and CDNC01000001 to CDNC01000051, respectively); and a human genitourinary T. phagedenis isolate (accession no. NZ_AEFH00000000).

### DNA isolations.

For collection of bacterial genomic DNA from OTEB cultures, 2 ml of culture was centrifuged (5,000 × *g*, 10 min, 4°C) in a bench-top centrifuge. DNA was then extracted from the cell pellet by using Chelex-100 as previously described (29) and was stored at −20°C.

### 16S rRNA gene PCR.

The 16S rRNA gene was amplified as described previously (10) from the 48 new isolates included in this study (Tables 1 to 3). Isolates were confirmed to contain only a single phylogroup by use of nested PCRs specific for the three unique treponeme phylogroups (10).

### Multilocus sequence typing.

The genetic loci used for this study were akin to those used for MLST of another pathogenic spirochete genus, Brachyspira (24).

The presence of a single copy of each locus within the genomes of representatives of each of the three DD treponeme phylogroups was confirmed by analysis of almost complete (>93%) genomes available online (for T. medium [accession no. KE332517.1], T. phagedenis F0421 [accession no. AEFH01000000], and T. pedis [CP004120]). Furthermore, the loci were identified as being well dispersed along these genomes (>100 kb between loci). Primers were designed to amplify fragments of genes encoding a heat shock protein (GroEL), recombination protein A (RecA), glycerol kinase (GlpK), adenosine kinase (AdK), glutamate dehydrogenase (GDH), orotidine 5′-phosphate decarboxylase (PyrG), and the large RNA polymerase subunit (RplB) by reference to the genome sequences described above, using Primer3 (30), such that all amplicons were 500 to 600 bp long (Table 4).

**TABLE 4:** PCR primers used to generate amplicons of housekeeping genes for MLST of the three treponeme phylogroups^*a*^

Locus	Treponeme group	Putative encoded protein	Predicted product size (bp)	Position^*b*^	Primer sequence (5′–3′)	
					Forward	Reverse
*groEL*	DD1	Heat shock protein	545	768883–769428	CTTGAATTAAAGCGCGGTATG	AAAATAGCGATATCTTCGAGCATT
	DD2	Heat shock protein	549	768883–769428	CTTGAGCTGAAACGAGGAATG	GGTAAGAATAGCAATATCTTCAAGCA
	DD3	Heat shock protein	542	768883–769428	GCTTGAATTAAAACGCGGAAT	CTGCAATATCTTCAAGCATTTCTTT
*recA*	DD1	Recombination protein A	571	2449887–2450338	CTACAAATCGAAAAGGAGTTTGGA	CGTACGCAATACCGATTTTCAT
	DD2	Recombination protein A	572	2449887–2450338	GCCTTCAAATCGAAAAACAATTC	GAACATAACGCCGATTTTCAT
	DD3	Recombination protein A	560	2449887–2450338	AAATTGAAAAACAATTCGGACAG	AACACCGATTTTCATTCTTATTTGA
*glpK*	DD1	Glycerol kinase	613	1797272–1797770	TATTTTATCATTCGATCAGGGAACA	AATATTCAGTTCCGTCAGAATTTCA
	DD2	Glycerol kinase	610	1797272–1797770	ATATTTTAGCACTTGATCAGGGAAC	CCGAGTTCTTGTAAAATCTCATCAT
	DD3	Glycerol kinase	589	1797272–1797770	ATCTTTTGACCAAGGAACTACAAGT	TAACTCATTATCCCATTCCAAAGTC
*adk*	DD1	Adenosine kinase	517	2265510–2265903	CTGCAAAATATTATGGTATCCCTCA	GCATCCAAAGTTATGAGCAGTTTT
	DD2	Adenosine kinase	499	2265510–2265903	GCTATCAAATCCCGCATATTTC	TTTGCGAGTACATTTTTCTTTTCAT
	DD3	Adenosine kinase	526	2265510–2265903	TCAAAGTTGTACAAGATACCGCATA	ATGAGGGACGTGCGTCAATA
*gdh*	DD1	Glutamate dehydrogenase	647	275169–275682	CGTCAATACTAACGGACAGATTATG	GGTTCTGTACCCATTCAAAGTAAGA
	DD2	Glutamate dehydrogenase	643	275169–275682	GTCAACACAAACGGGCAAATAAT	TCTGAACCCATTCAAAGTAAGAAAC
	DD3	Glutamate dehydrogenase	623	275169–275682	GTGGGTACAAATGCGAAAATTATG	CATTCAAAATACGAAACAATTACCC
*pyrG*	DD1	Orotidine 5′-phosphate dehydrogenase	601	2320945–2321441	CAGGTTATCCCGCATGTTACC	ACGCTTCGCTTACGCTTAAATAC
	DD2	Orotidine 5′-phosphate dehydrogenase	611	2320945–2321441	GTACAAGTTGTCCCGCATGTAAC	GCAGTCAGCGCTTCACTCAC
	DD3	Orotidine 5′-phosphate dehydrogenase	596	2320945–2321441	GTACCCCATGTAACCGATGAA	AGGGCTTCCACTACGCTTAAATA
*rplB*	DD1	Large polymerase subunit	565	953257–953715	ATATAAGCCTATAACACCGGGTATG	ACCGATTGTTGCATAGCATTTT
	DD2	Large polymerase subunit	575	953257–953715	ATAAGCCTATAACACCGGGACTAAG	ATTTCCAACTTCACCGATTGTC
	DD3	Large polymerase subunit	575	953257–953715	TCTAAAAGAATATAAGCCGATGACG	CGCCTATGGTAGCATAACATTTTT

a One primer set each was developed for the T. medium phylogroup (DD1), the T. phagedenis phylogroup (DD2), and the T. pedis phylogroup (DD3).

b Positions of genes correspond to those in T. vincentii OMZ 838 (GenBank accession no. CP009227).

PCR master mixes for each locus were set up as previously reported (10, 13), but incorporating the new MLST primers (Table 4). All PCRs were carried out using the following cycling conditions: 95°C for 1 min followed by 40 cycles of 95°C for 1 min, 55°C for 1 min, and 72°C for 2 min, with a final extension of 72°C for 10 min.

### Sequencing and sequence analysis.

Amplified PCR products were sequenced commercially (Macrogen, Amsterdam, the Netherlands), and the data for each locus were verified and assembled using the Chromas Pro 1.7.5 sequence analysis package (Technelysium Pty. Ltd.). Gene sequences were aligned using CLUSTALW as implemented in MEGA 5.0 (31). Alleles and sequence types (STs) were assigned manually and analyzed using eBURST (32; data not shown).

To infer a phylogeny from 16S rRNA gene data, an alignment of sequences was subjected to ModelTest as implemented in Topali (33), which revealed that the best-fitting model was a general time reversible (GTR) model. This was used to produce nucleotide maximum likelihood phylogenetic trees (with bootstrap values based on 10,000 iterations). For each isolate, sequence data for the seven MLST loci were concatenated, and concatenated data from different isolates were aligned. Phylogenetic inferences from this alignment were made as described above. Concatenated gene trees were drawn using TN93 models (34), and all maximum likelihood trees were produced using 10,000 bootstrap values. Minimum spanning distance trees were drawn using Prim's algorithm (56). Alignments were screened for evidence of recombination by use of SplitsTree4 (35) and for positive and negative selection by use of GARD and SLAC, available through the Datamonkey Web server (36).

## RESULTS

### 16S rRNA gene analysis.

Almost complete 16S rRNA gene sequences were obtained for the 48 new DD treponeme isolates obtained in the study. Phylogenetic inferences derived from these data and those for the other 73 isolates included in the study indicated that all could be accommodated within one of the three previously described DD treponeme phylogroups (see Fig. S1 in the supplemental material).

Thus, the study included 34 isolates belonging to the T. medium phylogroup (DD1), 70 isolates belonging to the T. phagedenis phylogroup (DD2), and 17 isolates belonging to the T. pedis phylogroup (DD3).

### MLST data.

For all 121 isolates, sequences were obtained for all seven MLST loci. Comparison of the sequence data revealed variation at all loci, with no cases of full gene recombination seen between any of the three phylogroups. The average dissimilarity between loci for the three different phylogroups was 28.46% (range, 17.9% [*groEL*] to 39.26% [*adk*]). Furthermore, all loci varied within phylogroups, with dissimilarities ranging from 0.5% (*adk* in the T. phagedenis phylogroup) to 17% (*adk* in the T. medium phylogroup) (Table 5). Sequence variation at loci was far more pronounced in the T. medium phylogroup (mean = 10.9%) than in the T. phagedenis (mean = 1.2%) or T. pedis (mean = 2.5%) phylogroup. Even when the outlying T. vincentii strain was excluded from the T. medium phylogroup, the mean sequence variation among the remaining members was 4.9% (Table 5).

**TABLE 5:** Analysis of individual genes^*a*^

Locus	T. medium (DD1) phylogroup (*n* = 33)					T. phagedenis (DD2) phylogroup (*n* = 71)					T. pedis (DD3) phylogroup (*n* = 17)				
	Amplicon size (bp)	No. (%) of variable sites (DNA), with [without] inclusion of T. vincentii	No. (%) of variable sites (aa) with [without] inclusion of T. vincentii	No. of DNA alleles	No. of aa alleles	Gene size (bp)	No. (%) of variable sites (DNA)	No. (%) of variable sites (aa)	No. of aa alleles	No. of DNA alleles	Gene size (bp)	No. (%) of variable sites (DNA)	No. (%) of variable sites (aa)	No. of DNA alleles	No. of aa alleles
*groEL*	448	40 (9) [15 (3)]	0 [0]	3	1	456	6 (1.3)	4 (3)	2	3	441	13 (3)	0	4	1
*recA*	475	64 (13) [12 (2)]	59 (37) [11 (7)]	3	3	472	12 (2.5)	4 (3)	3	9	477	10 (2)	0	6	3
*glpK*	507	34 (7) [20 (11)]	31 (7) [18 (10)]	4	4	521	4 (0.7)	3 (1.7)	4	5	508	5 (1)	5 (3)	4	4
*adk*	416	69 (17) [27 (6)]	57 (41) [23 (17)]	5	6	394	2 (0.5)	1 (0.7)	2	3	421	13 (3)	3 (2)	4	4
*gdh*	514	47 (9) [11 (6)]	7 (1) [2 (1)]	4	4	560	10 (1.8)	9 (5)	2	5	520	22 (4)	16 (11)	5	4
*pyrG*	501	52 (10) [21 (4)]	47 (28) [18 (11)]	4	4	527	5 (0.9)	0	1	3	507	21 (4)	0	5	2
*rplB*	469	54 (11) [8 (2)]	47 (30) [8 (5)]	4	4	475	3 (0.65)	2 (1)	2	2	502	10 (2)	0	4	4

a Gene sizes and allelic arrangements, at both the nucleotide and amino acid (aa) levels, are shown. Because T. vincentii appears to form a separate species, it was analyzed both in conjunction with and separately from the T. medium phylogroup.

The number of alleles for each locus ranged from 10 to 18, with a range of 2 to 9 for individual phylogroups (Table 5). Sequence types were assigned based on the MLST allelic profiles. Comparison of allelic profiles revealed a total of 53 STs: 11 within the T. medium phylogroup (Table 1), 35 within the T. phagedenis phylogroup (Table 2), and 7 within the T. pedis phylogroup (Table 3). Unique allelic sequences were obtained for each of the different phylogroups. Of 11 STs within the T. medium phylogroup, ST1 was encountered most frequently (14/34 [41%] isolates) (Table 1). However, no ST was so dominant in the other two phylogroups, suggesting that they contain greater sequence variation. In the T. phagedenis phylogroup (Table 2), ST16 was the most common, but only 6 of 67 (8%) isolates possessed this ST. In the T. pedis phylogroup, ST5 was the most common, but only 6 of 17 (35%) isolates possessed this ST (Table 3). Of the 53 total STs encountered, 29 were represented by only one isolate each.

**FIG 1:**
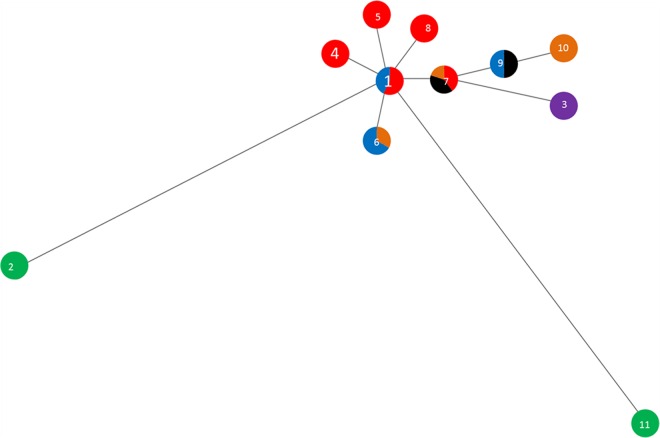
Minimum spanning distance tree for isolates of the T. medium phylogroup (DD1). Further details of the STs are shown in Table 1. Each ST circle is colored based on the proportion of the sequences within it which were isolated from each host. Numbers correspond to the ST numbers shown in Table 1. Red, dairy cow isolates; black, beef cow isolates; purple, goat isolates; orange, elk isolates; blue, sheep isolates; green, human isolates.

Minimum spanning trees compare similarities among different isolated STs and how closely related they are. Therefore, isolates located close to each other on a tree are generally different at one of the MLST loci, whereas more distant isolates have fewer loci in common. The T. medium phylogroup minimum spanning tree showed relationships centered around the founder ST, ST1 (Fig. 1), which contains both cattle and sheep isolates (Table 1). The T. vincentii OMZ 38 sequence type (ST11) and the human T. medium ATCC 700293 sequence type (ST2) were outliers in the data, further suggesting that they are profoundly divergent from DD-associated strains. These data also further corroborate that T. vincentii is not a member of the T. medium phylogroup but is a separate species (Fig. 1 and Table 1; also see Fig. 4).

**FIG 2:**
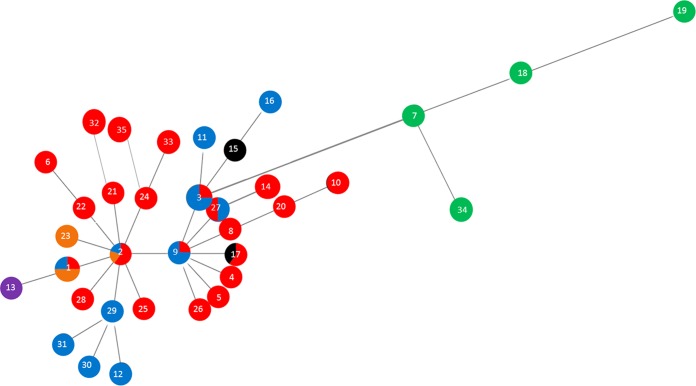
Minimum spanning distance tree for isolates of the T. phagedenis phylogroup (DD2). Further details of the STs are shown in Table 2. Each ST circle is colored based on the proportion of the sequences within it which were isolated from each host. The numbers correspond to the ST numbers shown in Table 2. Red, dairy cow isolates; black, beef cow isolates; purple, goat isolates; orange, elk isolates; blue, sheep isolates; green, human isolates.

Data for the T. phagedenis phylogroup minimum spanning distance tree (Fig. 2) suggested that ST2 was the founder ST, with nine other STs as single-locus variants (SLVs). However, the neighboring ST, ST9, possessed eight SLVs (Table 2 and Fig. 2). For both the T. medium (Fig. 1 and Table 1; also see Fig. 4) and T. phagedenis (Fig. 2 and Table 2; also see Fig. 5) phylogroups, human isolates were distant from the animal isolates.

**FIG 3:**
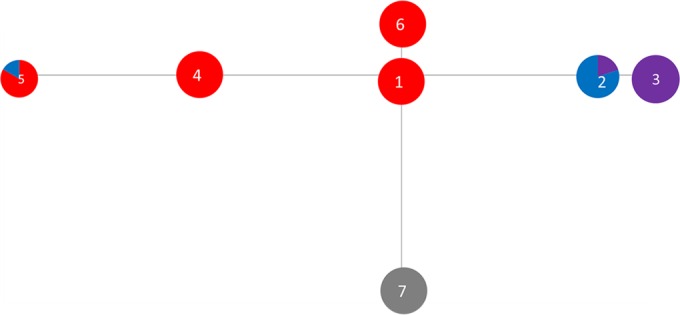
Minimum spanning distance tree for isolates of the T. pedis phylogroup (DD3). Further details of the STs are shown in Table 3. Each ST circle is colored based on the proportion of the sequences within it which were isolated from each host. The numbers correspond to the ST numbers shown in Table 3. Red, dairy cow isolates; black, beef cow isolates; purple, goat isolates; orange, elk isolates; blue, sheep isolates; gray, pig isolates.

The T. pedis phylogroup minimum spanning tree shows isolates radiating out from ST1 (which contains T3552B^T^) (Fig. 3). A larger amount of variation is seen within the T. pedis tree than within the trees for the other two phylogroups (Fig. 3 and Table 3; also see Fig. 6). The newer sequences (ST2 and ST3), isolated from sheep and goats, form a distinct cluster away from the older isolates, which were largely isolated from cattle.

All allelic data were uploaded into pubMLST (37).

### Molecular epidemiology.

**FIG 4:**
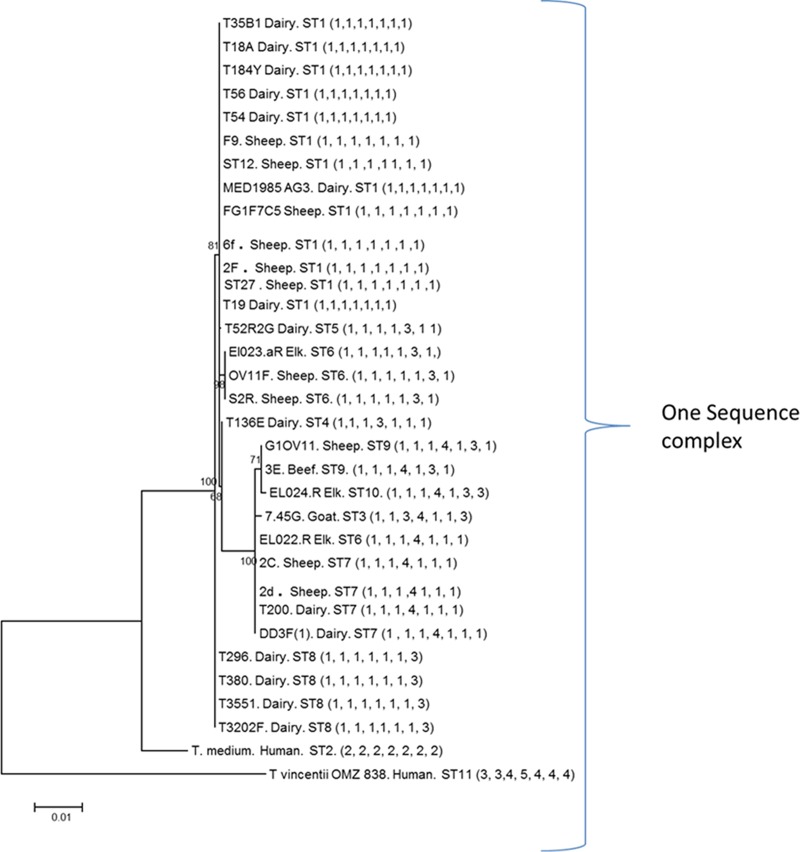
Concatenated gene DNA phylogenetic tree for seven housekeeping genes for the T. medium phylogroup (DD1). Each bacterium is labeled with the isolate name, the host from which it was isolated (dairy or beef cow, sheep, goat, elk, or human), the ST to which it belongs (Table 1), and the allelic arrangement for that isolate (in parentheses).

Many STs in all three phylogroups were encountered in more than one host species and in multiple geographic locations. Within the T. medium phylogroup, four of the five STs (STs 1, 6, 7, and 9) that contained more than one isolate were recovered from different host species (Fig. 4). In the case of ST1 of the T. medium phylogroup, these isolates were from both cattle and sheep. Additionally, three of these T. medium phylogroup STs contained isolates recovered from animals inhabiting geographically distant countries, including ST1 being present in England, Wales, and the United States. Conversely, we also obtained isolates belonging to different STs of the same phylogroup from the same host species on the same farm (Table 1).

In contrast, the human T. medium ATCC 700293 and T. vincentii OMZ 838 isolates had unique allelic arrangements.

**FIG 5:**
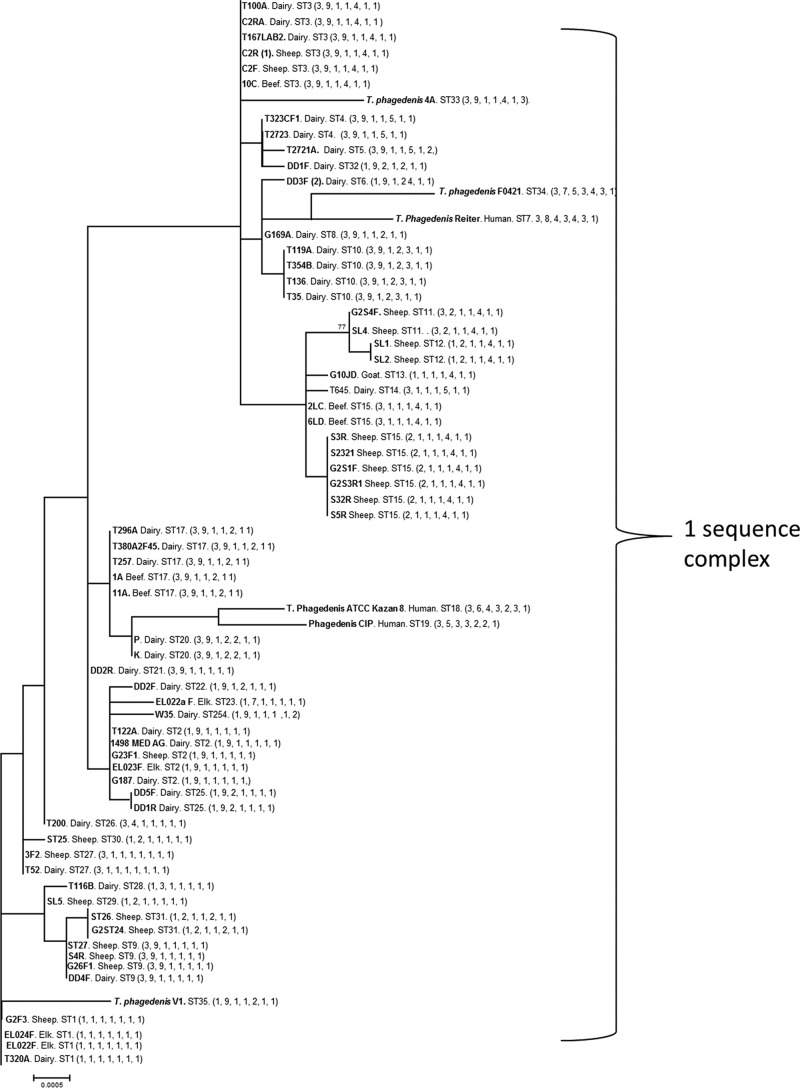
Concatenated gene DNA phylogenetic tree for seven housekeeping genes for the T. phagedenis phylogroup (DD2). Each bacterium is labeled with the isolate name, the host from which it was isolated (dairy or beef cow, sheep, goat, elk, or human), the ST to which it belongs (Table 2), and the allelic arrangement for that isolate (in parentheses).

Within the T. phagedenis phylogroup, similar patterns were seen, with 6 of the 15 STs (STs 1, 2, 3, 9, 17, and 27) which contained more than a single isolate being recovered from different host species (Table 2 and Fig. 5).

Twenty of the 35 T. phagedenis phylogroup STs were singletons, containing only one isolate. As with the T. medium phylogroup, all four human isolates of T. phagedenis had unique allelic arrangements (STs 7, 18, 19, and 34) (Fig. 5 and Table 2).

**FIG 6:**
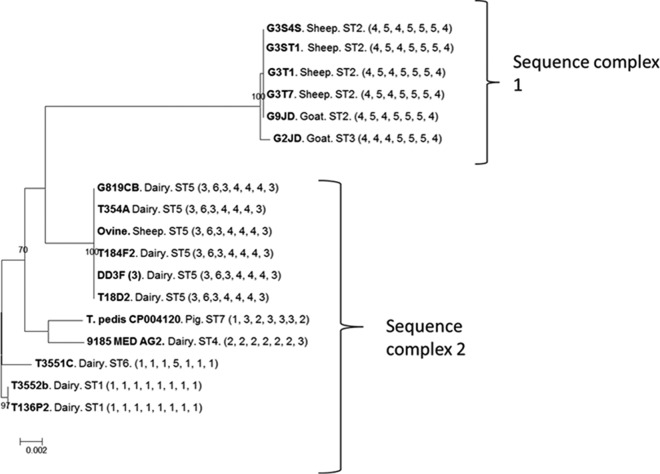
Concatenated gene DNA phylogenetic tree for seven housekeeping genes for the T. pedis phylogroup (DD3). Each bacterium is labeled with the isolate name, the host from which it was isolated (dairy or beef cow, sheep, goat, or pig), the ST to which it belongs (Table 3), and the allelic arrangement for that isolate (in parentheses).

Although T. pedis isolate numbers were smaller, two (ST2 and ST5) of three STs that contained more than a single isolate were recovered from different host species (Table 3 and Fig. 6).

Of the 19 farms used in this study, 13 had isolates circulating on them that belonged to more than one ST (Tables 1 to 3).

### Evolutionary features within loci.

Nucleotide polymorphisms were seen in all loci tested for all three of the DD treponeme phylogroups. Within some loci, there appeared to be regions of sequence in which single nucleotide polymorphisms (SNPs) were concentrated. For example, among T. pedis phylogroup members, 12 of the 13 SNPs in a 421-bp *adk* PCR product occurred in the final 150 bp of the locus. Similarly, among T. phagedenis phylogroup members, 7 of 10 SNPs in a 560-bp *gdh* PCR product occurred in a 30-bp section from nucleotides 464 to 494.

Analysis of the data for each locus did not reveal any evidence of positive selection pressures, although among the T. medium phylogroup members, sites within the *adk*, *pyrG*, and *rplB* loci appeared to be under negative or purifying pressure (see Table S1 in the supplemental material).

Split decomposition analysis suggested that, in general, recombination has had a marked influence on the divergence of STs within all three phylogroups (see Fig. S2 in the supplemental material). However, we were unable to find evidence of recombination between different phylogroups (data not shown).

### Phylogeny.

In concurrence with the phylogeny inferred from alignment of 16S rRNA gene sequence data, the phylogeny inferred from alignment of the concatenated MLST locus sequence data divided the DD treponemes investigated in this study into three deeply diverging phylogroups. Both the T. medium (Fig. 4) and T. phagedenis (Fig. 5) phylogroups form single sequence complexes. However, the T. pedis phylogroup has diverged into two different sequence complexes, designated based on similarity to the central allelic profile (in this case, ST1) (Fig. 6).

## DISCUSSION

The recent expansion in the host range of DD Treponema spp. to include a variety of additional food chain animals has led to a greater number of animal welfare issues and greater substantial economic losses to agricultural industries (4, 5, 7–13, 18, 38–40). Furthermore, the inter- and intra-host-species spread of these bacteria needs to be given special consideration, as isolates from humans and all animal species are considered to be very similar or identical (4, 5, 7).

Therefore, the use of a treponeme isolate archive in this study created a relatively unique opportunity to study bacterial species that can infect and cause disease in multiple animal species. As MLST analyses have previously been used to clarify relationships within a bacterial species and to differentiate bacteria by host species (24, 41), MLST was used in an attempt to differentiate DD treponemes isolated from different host species.

In this study, a collection of 121 DD Treponema isolates from nine different countries and three different continents were analyzed by MLST to elucidate the relationships between isolates from different host species, but the collection was limited by the geographic ranges of species (e.g., elk) and diseases (e.g., contagious ovine digital dermatitis [CODD]). That said, this is the largest and most rigorous molecular genetic analysis of DD treponemes isolated from humans and animals.

### Cultivable DD treponemes can be classified into three distinct phylogroups.

All cultivable DD treponeme isolates included within this study fit into the three previously reported phylogroups (10, 14), except for the human periodontal disease-associated T. vincentii isolate, which was unique at each locus tested, suggesting that it belongs to a different phylogroup and is unrelated to any farm animal disease-associated isolates despite high 16S rRNA gene similarities.

The analyses of 16S rRNA and housekeeping gene loci of currently isolated DD treponemes confirmed their classification into the three previously designated phylogroups: the Treponema medium, Treponema phagedenis, and Treponema *pedis* phylogroups (4, 5, 10, 11, 13, 14, 18, 19).

Sequence analysis of seven DD treponeme housekeeping genes revealed a generally low level of diversity among the strains within each phylogroup, removing the need for the previously used “-like” suffix. Taking the data together, we recommend removal of the “-like” suffix and instead refer to the bacteria as belonging to a phylogroup, such as the T. medium phylogroup, in line with similar studies of pathogenic mycobacteria (42). This was also recently suggested for T. phagedenis isolates (17).

Although phylogenetic, eBURST, and minimum spanning tree analyses revealed limited data regarding evolutionary relationships in clonal complexes (33, 43), together these approaches show that all isolated treponemes in this study group into three unique phylogroups, suggesting that they have different evolutionary lineages but a common ancestor. They also show that the T. pedis phylogroup is beginning to form two distinct ST complexes, based on related MLST allelic arrangements, with the newer isolates separating from the older isolates. This raises the importance of continued surveillance and vigilance of DD treponeme infections, as emergence of a new species may lead to an increased pathogenicity and, potentially, host range. Isolation of members of the T. pedis phylogroup appears to be less common (or successful) than that for the other two phylogroups, as only 17 members were isolated and analyzed in this study, compared to 34 T. medium and 70 T. phagedenis phylogroup treponemes. Isolation of more T. pedis phylogroup treponemes in the future will further help to delineate the two ST complexes which this phylogroup appears to be forming. However, the overall variation within the phylogroup is limited, with isolates from pigs, cattle, sheep, and goats all being relatively similar.

Although variation is seen within each locus for the T. medium and T. phagedenis phylogroups, including the 16S rRNA gene, the loci all group phylogenetically, and both phylogroups form single clonal complexes.

### Cultivable DD treponemes show limited genetic variability within phylogroups.

Identical bacteria were isolated from different host species, and 12 of the 23 sequence types with more than a single isolate in them were from different host species, such as with T. phagedenis ST1. This contrasts with the situation for other clinically significant spirochetes, such as Brachyspira spp., where isolates from different hosts generally belong to different bacterial species (24). Furthermore, STs within several different species of Leptospira are generally separated by host and geography (20), while geographic separation of Borrelia burgdorferi strains between two locations in the United States can clearly be identified (44). Therefore, this study demonstrates that MLST may not be suitable for differentiation of cultivable DD treponemes isolated from different host species, or it might be considered that the inability to discriminate identifies the occurrence of frequent transmission events between host species. Alternatively, it may be that the limited geographic sampling and relatively small isolate numbers included in this study make differentiation by MLST difficult.

Indeed, all genes sequenced here, from all three phylogroups, showed relatively little diversity, suggesting that the bacteria potentially have evolved genes which are highly functionally fit and are under little selection pressure to evolve further. However, among the three phylogroups, T. pedis was the most diverse. Previous studies suggested that some sections of the T. pedis genome have been lost compared to that of its closest relative, T. denticola, which further suggests that it is evolving rapidly (7). This increased evolution rate may agree with reports that T. pedis is more surface dwelling (45, 46) than members of the other phylogroups and therefore is likely to have to adapt to more rapidly alternating conditions, resulting in increased genetic diversity compared to that of deeper-tissue dwellers (45, 46).

Treponema pallidum, the causative agent of syphilis and yaws, shows a low level of diversity despite multiple isolations over many years, and it is highly similar to the related bacterium, Treponema paraluiscuniculi, the causative agent of rabbit venereal spirochetosis (47). These bacteria are similar or identical at the 16S rRNA gene level, but they infect two very different hosts. The data presented here show that the animal and human cultivable DD treponeme phylogroups have an even greater capacity to infect numerous hosts while undergoing little genetic alteration and evolution.

### Treponemes evolve by within-phylogroup recombination.

This study showed both recombination and some negative selection within DD treponeme phylogroups, unlike the observations for T. denticola (48). Treponema denticola is monophyletic, as are the three DD phylogroups in this study. However, in this study, the T. pedis phylogroup was more variable, diverging into two separate ST complexes, suggesting a more rapid evolution than that of the other phylogroups. Recombination was seen within the DD treponeme phylogroups but not between phylogroups, as evidenced by the lack of cross-reactivity between primers, and this was further confirmed by the split decomposition analysis. The use of different oligonucleotides for amplification of the different phylogroups further supports the continued usage of the three unique groupings of culturable treponemes suggested previously (10). Similar issues were identified in previous studies using Brachyspira spp. and Campylobacter spp., where it was reported that it is difficult to develop MLST oligonucleotides to amplify genes from members of an entire genus (24, 41).

### Bacterial spatial dynamics reveal multiple transmission events both locally and globally.

Within each DD treponeme phylogroup, limited evidence of a correlation between genotype and geographic provenance was seen. In some cases, STs were concentrated on a single farm or a few localized farms (e.g., ST16 in the T. phagedenis phylogroup), whereas others were found in different areas of the country (e.g., ST5 of the T. pedis phylogroup), and some were more global (e.g., ST7 from the T. medium phylogroup). This suggests that STs can spread and circulate worldwide among different animals. This is in contrast to Borrelia species, which show a geographic delineation, with European and American isolates being phylogenetically separate (22, 49).

The spatial dynamics of the bacterial STs suggest that identical bacteria can circulate on a farm, spreading around a flock or herd, such as was seen for T. medium phylogroup ST1 and ST7, T. phagedenis phylogroup ST16, and T. pedis phylogroup ST2. On-farm spread was more apparent among sheep flocks than in cattle herds, possibly due to closer confines and higher stocking densities of sheep. Additionally, the clinical manifestation of the disease causes much greater morbidity in sheep than in cattle, so it may be that cattle appear asymptomatic, whereas sheep show clinical signs more quickly and more noticeably. Many cattle farms also appear to be infected endemically, whereas sheep farms tend to present with episodic epidemics.

Other bacterial species, such as ST1 in the T. medium phylogroup, ST1 in the T. phagedenis phylogroup, and ST5 in the T. pedis phylogroup, can infect multiple host species, increasing their transmission. Furthermore, the similarities between isolates from different animal hosts raise the possibility of both inter- and intra-host-species transmissions, but the mechanism for this spread remains unclear.

In future, comparative analyses of full DD treponeme genomes isolated from a range of hosts will further delineate whether the same treponemal strains are indeed responsible for the recent expansion in host range and pathology, in line with the results from the current study. Such studies will also increase our knowledge of pathogen evolution and disease transmission to better inform farm practice, prevent severe diseases, and enhance global food security.

## Supplementary Material

Supplemental material
