# Supplementary material for: Multilocus Sequence Typing of Pathogenic Treponemes Isolated from Cloven-Hoofed Animals and Comparison to Treponemes Isolated from Humans
Source: Appl Environ Microbiol. 2016 Jul 15;82(15):4523–36. doi: 10.1128/AEM.00025-16 (PMC4984274; doi:10.1128/AEM.00025-16)
Supplement: Supplemental material [file supp_82_15_4523__index.html]

Multilocus Sequence Typing of Pathogenic Treponemes Isolated from Cloven-Hoofed Animals and Comparison to Treponemes Isolated from Humans — Supplemental material 

# Multilocus Sequence Typing of Pathogenic Treponemes Isolated from Cloven-Hoofed Animals and Comparison to Treponemes Isolated from Humans

## Supplemental material

- Supplemental file 1 -

  Treponeme phylogenetic tree based on 16S rRNA gene sequences (Fig. S1), split graphs showing recombination between isolates from the three phylogroups examined in this study (Fig. S2), and analysis of selection pressures on individual genes (Table S1).

  PDF, 527K
