# Supplementary material for: Multilocus Sequence Typing of Pathogenic Treponemes Isolated from Cloven-Hoofed Animals and Comparison to Treponemes Isolated from Humans
Source: Appl Environ Microbiol. 2016 Jul 15;82(15):4523–36. doi: 10.1128/AEM.00025-16 (PMC4984274; doi:10.1128/AEM.00025-16)
Supplement: Supplemental material [file AEM.00025-16_zam999117294so1.pdf]

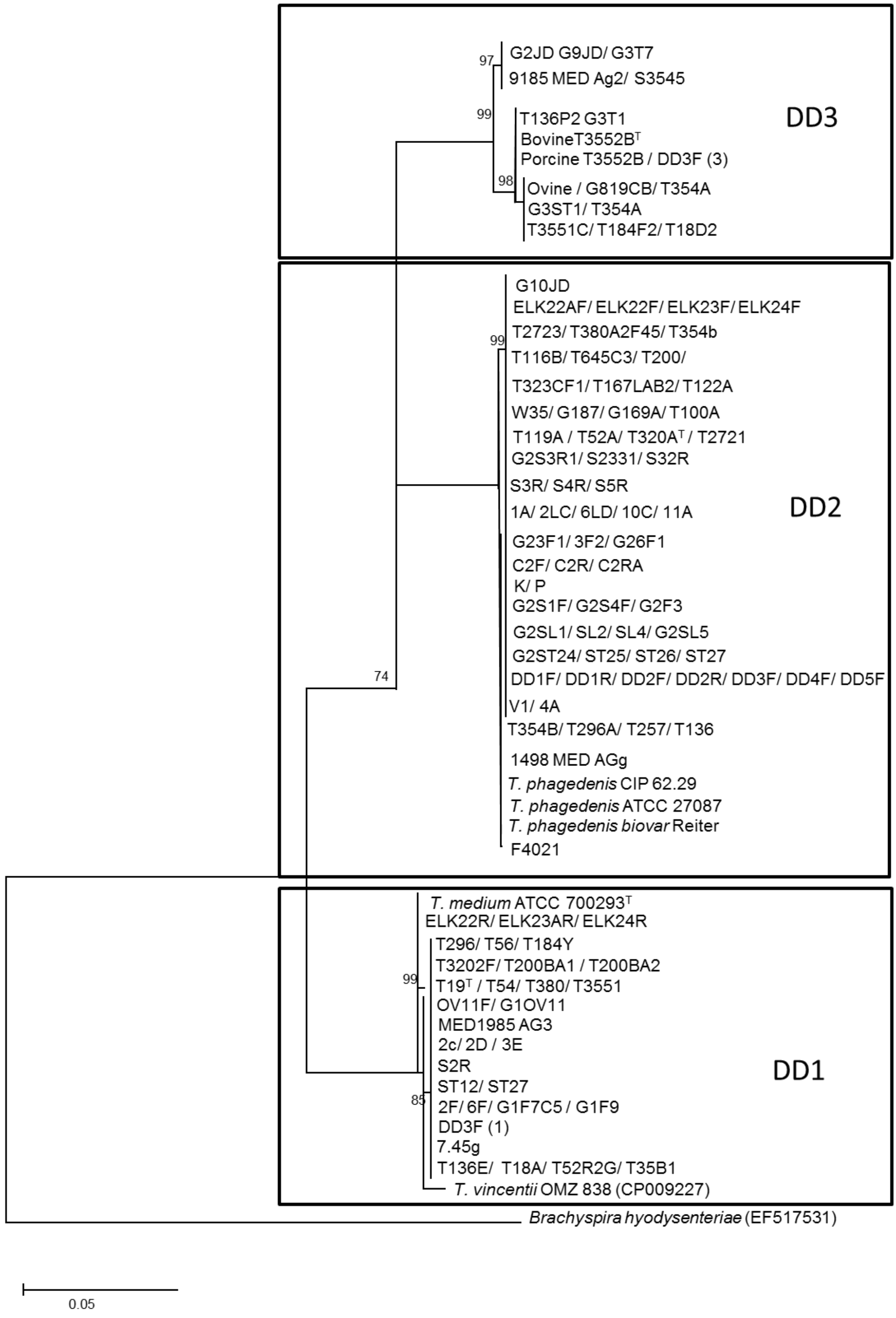

Figure S1. Treponeme phylogenetic tree based on 16S rRNA gene sequences. A maximum likelihood tree (bootstrapped 10,000 times) for comparison of the 16S

rRNA gene of the treponeme sequences isolated in this study. (For clarity, bootstrap values below 65 were removed). Genbank numbers for each individual isolate can be found in Tables 1, 2 and 3. For simplicity, due to the large number of samples in this study, sequences which have an identical 16S rRNA gene and from the same host species have been grouped together.

Key: DD1, DD2 and DD3 refer to the DD treponeme phylogroups, where DD1 is *Treponema medium*/*Treponema vincentii* phylogroup, DD2 is *Treponema phagedenis* phylogroup and DD3 is *T. pedis* phylogroup.

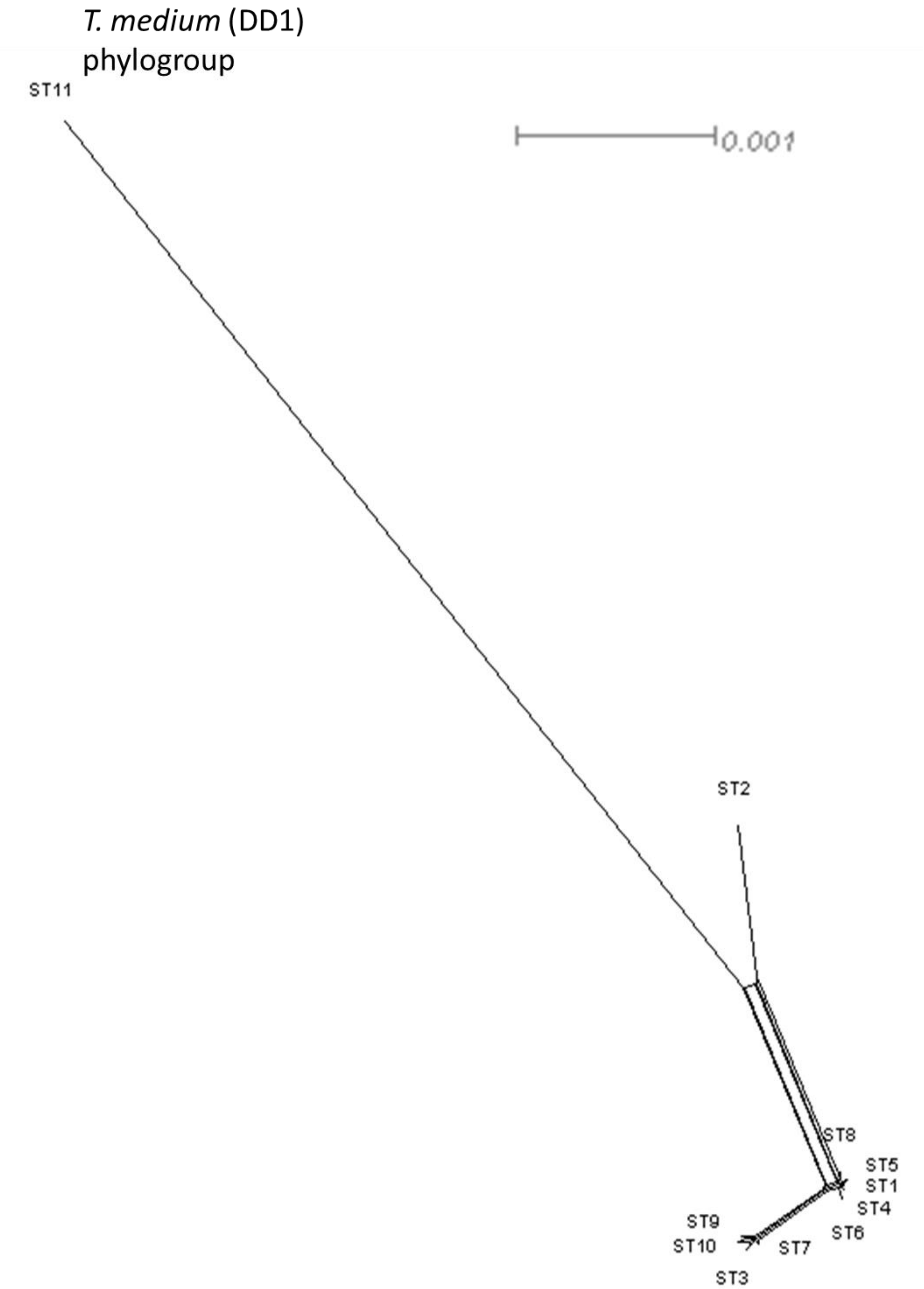
T. medium (DD1) phylogroup
ST11
0.001
ST2
ST8
ST5
ST1
ST4
ST6
ST9
ST10
ST7
ST3

T. phagedenis (DD2) phylogroup

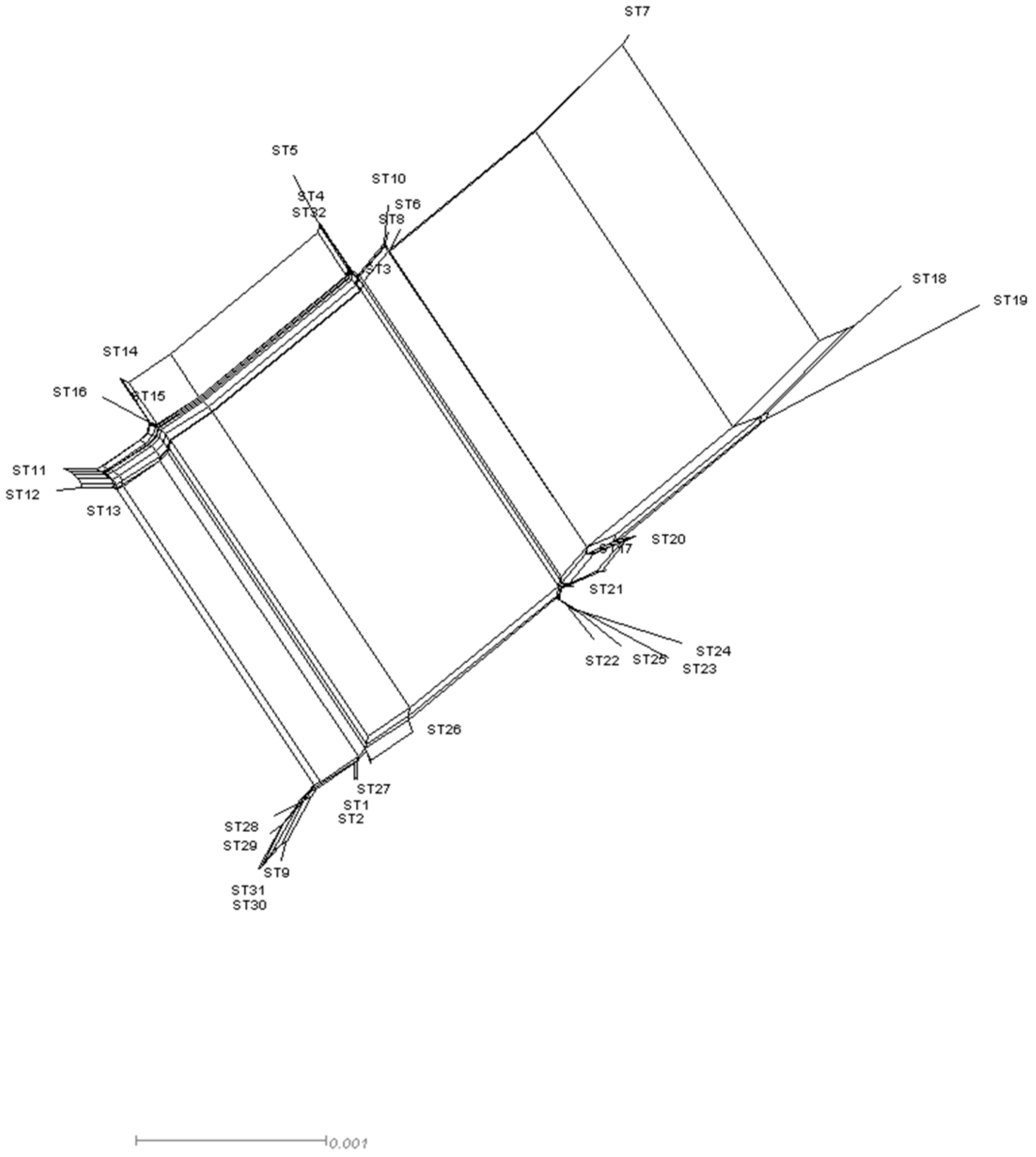

T. pedis phylogroup

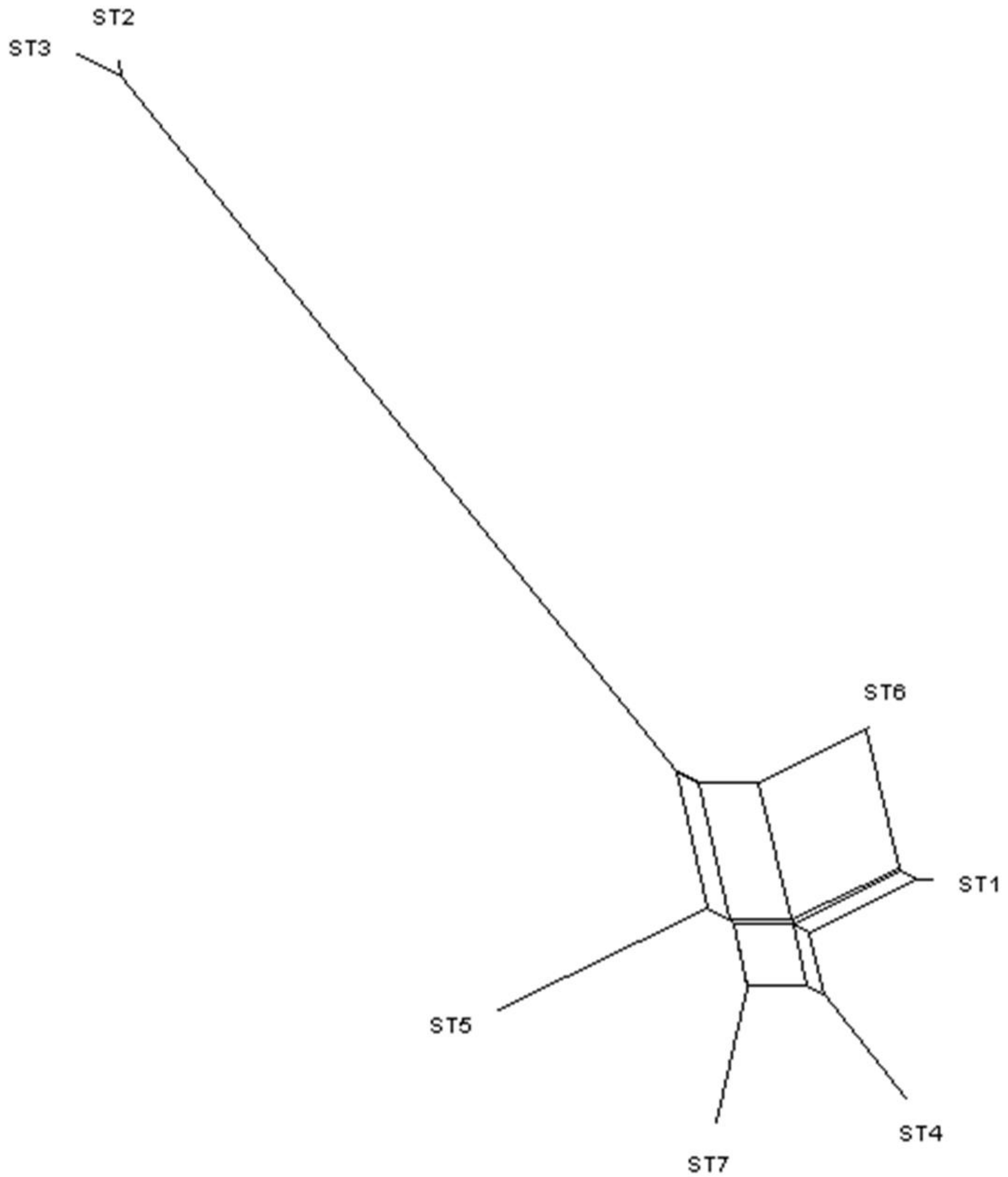

Figure S2. Splits graphs showing recombination between isolates from the three phylogroups examined in this study. Key: DD1, DD2 and DD3 refer to the DD treponeme phylogroups, where DD1 is *Treponema medium* phylogroup, DD2 is *Treponema phagedenis* phylogroup and DD3 is *T. pedis* phylogroup.

locus	*T. medium* (DD1) phylogroup (n= 34)			*T. phagedenis (DD2)* phylogroup (n= 70)			*T. pedis* (DD3) phylogroup (n=17)		
	Number of positive selection sites	Number of negative selection sites	d_N/d_S	Number of positive selection sites	Number of negative selection sites	d_N/d_S	Number of positive selection sites	Number of negative selection sites	d_N/d_S
groEL	0	0	5.000E-09	0	0	6.950E-01	0	0	5.000E-09
recA	0	0	5.238E-03	0	0	1.682E-01	0	0	2.782E-02
glpK	0	0	7.409E-02	0	0	7.269E-01	0	0	5.000E-09
adK	0	2 (86, 92)	7.974E-02	0	0	2.532E-01	0	0	1.168E-01
gdh	0	0	5.639E-02	0	0	4.386E-02	0	1 (162)	8.778E-02
pyrG	0	2 (91, 96)	3.206E-02	0	0	1.094E-01	0	1 (142)	5.000E-09
rplB	0	1 (78)	1.071E-02	0	0	5.000E-09	0	1 (110)	5.000E-09

Table S1. Analysis of selection pressures on individual genes**.** d_N/d_S is the ratio of non-synonymous substitutions per non-synonymous site to synonymous substitutions per synonymous site.
